# Prognostic value of decreased tongue strength on survival time in patients with amyotrophic lateral sclerosis

**DOI:** 10.1007/s00415-012-6503-9

**Published:** 2012-04-24

**Authors:** J. G. Weikamp, H. J. Schelhaas, J. C. M. Hendriks, B. J. M. de Swart, A. C. H. Geurts

**Affiliations:** 1 Department of Rehabilitation (898), Nijmegen Centre for Evidence Based Practice, Radboud University Nijmegen Medical Centre, P.O. Box 9101, 6500 HB Nijmegen, The Netherlands; 2 Department of Neurology (935), Donders Institute for Neuroscience, Radboud University Nijmegen Medical Centre, P.O. Box 9101, 6500 HB Nijmegen, The Netherlands; 3 Department of Biostatistics, Epidemiology and HTA (133), Radboud University Nijmegen Medical Centre, P.O. Box 9101, 6500 HB Nijmegen, The Netherlands

**Keywords:** ALS, Tongue strength, Survival, Prognosis

## Abstract

Decreased tongue strength (TS) might herald bulbar involvement in patients with amyotrophic lateral sclerosis (ALS) well before dysarthria or dysphagia occur, and as such might be prognostic of short survival. The purpose of this study was to investigate the prognostic value of a decreased TS, in addition to other prognostic factors, such as site of onset, bulbar symptoms, bulbar signs, age, sex, maximum phonation time, time from symptoms to diagnosis, and gastrostomy, for survival time in patients with ALS. TS was measured in four directions in 111 patients who attended the diagnostic outpatient motor neuron clinic of our university hospital. Of these patients, 54 were diagnosed with ALS. TS was considered abnormal if the strength in minimally one direction was at least two standard deviations below the reference values obtained from comparable age category and sex-groups of healthy controls (*n* = 119). Twenty of the patients with ALS had a decreased TS. Multivariable analysis showed that, in addition to age, TS was an independent prognostic factor for survival time in patients with ALS.

## Introduction

Amyotrophic lateral sclerosis (ALS) is a progressive disorder caused by degeneration of both upper (UMN) and lower (LMN) motor neurons in the bulbar and spinal regions. The cause of ALS is not known and to date there is no curative treatment. Knowledge of disease progression and survival time is of utmost importance for clinical care and research. The mean survival time is 3–5 years, although a small minority of patients (7 %) may survive for 10 years or longer [1, 6, 7, 15, 27]. Several prognostic factors for survival have been identified, such as age at diagnosis or onset, time between first symptoms and first examination, and site of symptom onset [6, 9, 11].

Survival is worse in patients with bulbar-onset ALS as compared with spinal-onset ALS [6, 27, 28]. Moreover, survival is significantly shorter in patients with bulbar involvement at first assessment independent of the site of onset [11, 27]. However, the strength of the association between bulbar involvement and survival time might be influenced by the way bulbar involvement is defined. Until now, most studies have described bulbar involvement as the presence of symptoms or signs of dysarthria or dysphagia. While studies have used questionnaires [7] and quantitative bulbar measurements [2, 12, 18, 19, 21] to describe bulbar changes, as far as we know an objective noninvasive quantitative measure of bulbar involvement has not been used to predict survival in ALS.

Tongue strength (TS) has been found to be a reliable indicator of bulbar involvement in various diseases [3, 13, 17, 20, 22, 24, 26] and in animal models of ALS [8]. Reduced TS in ALS might be caused by both UMN and LMN degeneration and can occur in patients with and without clinical symptoms of dysarthria or dysphagia [12, 19]. Bulbar muscle weakness in ALS is usually more pronounced in the muscles of the tongue than in muscles of the lips and jaw [12, 19]. The reason for this is not known but might be due to anterograde degeneration and the relatively large representation of the tongue in the primary motor cortex. Another possible explanation is that the motor units of the tongue function near their maximum firing rate under normal conditions, so compensatory additional activation might not be possible [21].

In the present study, we prospectively studied the prognostic value of a decreased TS compared to other known prognostic factors for survival in ALS. TS was assessed with a newly developed tongue force measurement device.

## Methods

### Participants

All patients who were referred to the diagnostic outpatient motor neuron clinic of the Radboud University Medical Centre (Nijmegen, the Netherlands) between October 2003 and June 2007 were eligible (*n* = 111). Referral was based on the medical suspicion of some form of motor neuron disease. Patients were included prospectively provided that a TS measurement could be performed on the same day as the neurological examination and the diagnostic electromyography (EMG) investigation. An experienced neurologist (HJS) diagnosed the patients and determined the site of disease onset on the basis of the first symptoms. TS was measured by an experienced speech and language therapist (JW). The maximum phonation time (MPT) was measured to estimate vital capacity [11, 16], because with this measurement it is also possible to assess voice quality. On a single breath, patients were instructed to say /a:/ for as long as possible. This procedure was repeated three times and the maximum sustained phonation time was used for analysis. The following data were recorded at time of the initial assessment: age, sex, site of onset, bulbar symptoms, bulbar signs, MPT, and TS.

Only patients with ALS [4] (‘possible’, ‘probable laboratory supported’, ‘probable’, or ‘definite’ according to the revised El Escorial criteria) at initial diagnosis or during follow-up were included in this study (*n* = 54). All patients were under the care of multidisciplinary rehabilitation teams specialized in ALS. Time from symptoms to diagnosis, date of gastrostomy placement, and time to death (if applicable) were collected during follow-up. When necessary, survival status and gastrostomy placement were determined by telephone calls.

Over the same time period, 119 healthy volunteers (aged 14–85 years) were asked to participate to obtain normal reference values for TS [25], with careful dividing into three age categories and sex groups (20 persons per group). The volunteers were recruited in the academic medical center (colleagues and partners of patients), from the personal environment (relatives and acquaintances), and after advertising at swimming and bowling centers. Exclusion criteria were a history of a neurological disease, other diseases that might cause decreased oropharyngeal strength (e.g., severe diabetes mellitus, chronic obstructive pulmonary disease, or otolaryngologic problems), the absence of teeth, and poorly fitting dentures.

The local ethics committee approved the study and all participants provided informed consent.

### Tongue strength

#### Transducer

**Fig. 1:**
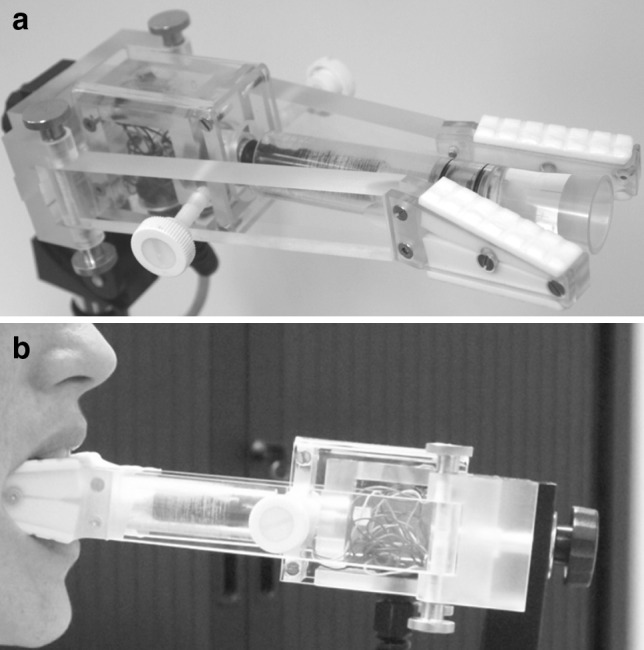
**a** Tongue strength measurement device. **b** The tongue strength measurement device in use

The TS transducer used (Fig. 1a) was developed on the basis of an earlier model [23, 26]. It consisted of a lever on which four strain gauges were arranged in a Wheatstone bridge configuration. The device was positioned between the teeth, and the tongue was placed in a cup at the end of the lever, and measurements in cranial, lateral, and frontal directions were taken, involving intrinsic as well as extrinsic tongue muscles. The left and right parts of the mouthpiece of the transducer were covered with impression material for stability and to ensure that the device was placed in the same position in the mouth of each participant at each measurement. The TS transducer was calibrated several times and values remained stable during the study period.

#### Measurement

All participants were assessed in the same way. Before the first measurements, they were carefully instructed and guided through several practice trials. Participants were seated on a normal chair and were asked to put the mouthpiece of the device in their mouth, to hold the bite fixation parts between their teeth, and to push the tongue against the transducer in the different directions (Fig. 1b). Once we had established that they understood the task to be performed, they were requested to push as hard as possible in a specific direction for ~5 s. This was repeated for every direction: upward, frontal, left, and right. One set of four directions was followed by a rest period of 30 s, to allow the muscles to relax. Then three other sets of measurements were taken, with a balanced sequence of the four force directions to exclude a direction effect due to fatigue. The mean values of the four sets in each direction were used as a measure of TS.

### Statistical methods

A TS score in at least one of the four directions of two standard deviations (SD) below the mean of the age- and sex-specific healthy reference group was considered abnormal. Fisher’s exact test was used to test differences between patients with and without an abnormal TS for statistical significance in the case of nominal variables (onset site, bulbar symptoms, bulbar signs, sex, gastrostomy, MPT) and Mann–Whitney *U* in the case of continuous variables (age and time from symptoms to diagnosis). For MPT, patients were allocated to two groups (MPT ≤10, MPT >10 s), because this threshold was found to be of clinical relevance [16].

Time from diagnosis to death was the primary outcome. The end-of-study date (August 1, 2011) was used for surviving patients and the date of last contact for the one patient who was lost to follow-up. Kaplan–Meier estimates are presented. The log rank test was used to test differences between the two TS groups with regard to time to death.

Univariable proportional hazard model was used to test the differences in survival for each independent variable separately (onset site, bulbar symptoms, bulbar signs, age, sex, MPT, TS, time from symptoms to diagnosis, and gastrostomy). The dependent variable was time to death. Crude hazard ratios (HR) with 95 % confidence interval (CI) are presented. A multivariable proportional hazard model was used with selection procedures to study the independent contribution of a decreased TS. The dependent variable was time to death. All variables in the univariable model with a *p* < 0.10 were valid for selection: TS, age, and onset site. The adjusted HR with 95 % CI of the final model are presented.

All statistical analyses were performed using SPSS, version 16.0 for windows (SPSS, Chicago, IL, USA).

## Results

Fifty-four of the 111 eligible patients were diagnosed with ALS and included in this study (25 males, age 20–76 years; 29 females age 45–80 years). The clinical and demographic characteristics of these patients are presented in Table 1.

**Table 1:** Clinical and demographic characteristics of the 54 patients with amyotrophic lateral sclerosis

	Tongue strength				*p*
	Abnormal (*n* = 20) median (range)/ *n* (%)		Normal (*n* = 34) median (range)/ *n* (%)		
Sex (male)	7	(35)	18	(51)	0.26*
Onset site (bulbar)	13	(65)	6	(18)	<0.00*
Bulbar symptoms (presence)	17	(85)	9	(32)	<0.00*
Bulbar signs (presence)	15	(75)	5	(15)	<0.00*
Gastrostomy (presence)	12	(60)	14	(41)	0.09*
MPT (<10 sec)^a^	6	(35)	5	(17)	0.28*
Age at diagnosis (year)	66.0	(20.0–80.0)	63.5	(40.0–79.0)	0.52**
Time from symptoms to diagnosis (month)	12.0	(6.0–60.0)	10.0	(4.0–60.0)	0.70**

^a^maximum phonation time in abnormal *n* = 17, normal *n* = 29

*p* Fisher’s exact test (*) and test of Mann–Whitney (**)

Twenty patients (37 %) had an abnormal TS. Of these, 13 patients (65 %) had a bulbar onset and 13 patients (65 %) were female. Gastrostomy was performed in 26 of the 54 patients (48 %) and in 12 (60 %) of the patients with an abnormal TS. The site of onset, the presence of bulbar symptoms, and bulbar signs were significant differences between patients with and without an abnormal TS.

Forty-five patients died during the study, 8 patients were still alive at the end of the study (one of whom had an abnormal TS at diagnosis), and 1 patient was lost to follow-up.

**Fig. 2:**
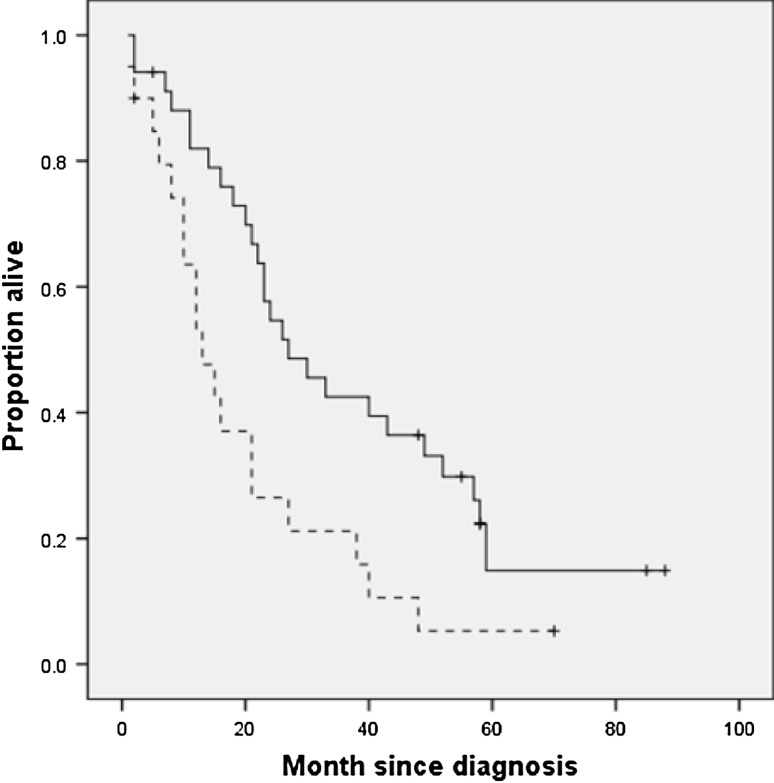
Kaplan–Meier estimates for time to death in patients with a normal tongue strength (TS; *solid line*) or an abnormal TS (*broken line*). *Vertical bars* indicate censured data. The *p* value for differences between TS groups was 0.01 using the log rank test

**Fig. 3:**
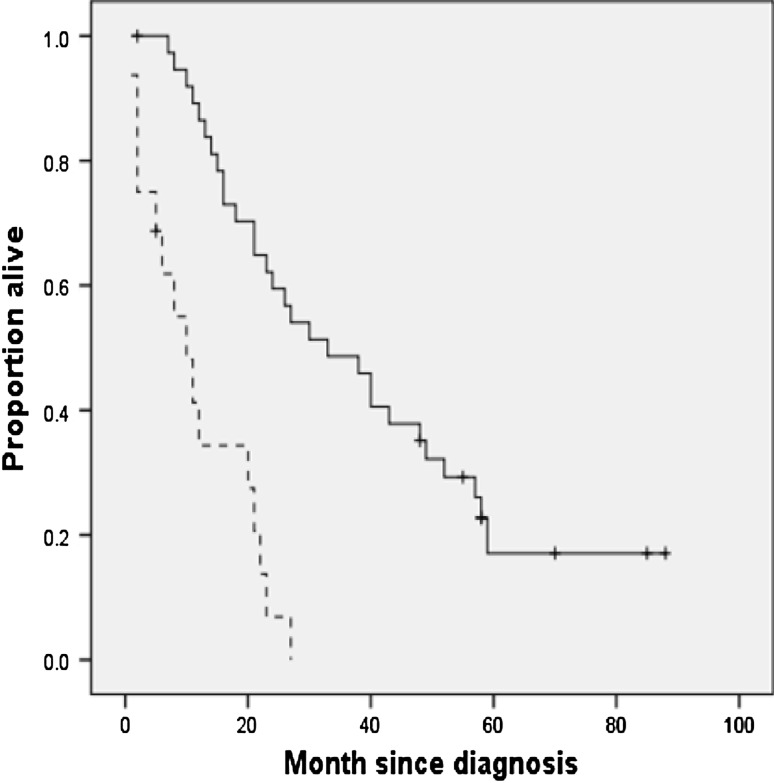
Kaplan–Meier estimates for time to death in patients younger (*solid line*) and older (*broken line*) than 69 years. *Vertical bars* indicate censured data. The *p* value for differences between the two age groups was <0.01 using the log rank test

The reference group of healthy volunteers consisted of 58 men and 61 women aged 14–85 years. Table 2 shows the age- and sex-specific reference values for TS in the left direction and the cut-off values (i.e., age and sex specific means −2 SDs). Reference values for the other directions (right, cranial, and frontal) are available from the first author. Kaplan–Meier estimates of time to death in patients with an abnormal and normal TS are shown in Fig. 2 and those in patients younger and older than 69 years are shown in Fig. 3. Table 3 shows the crude HR (95 % CI) for time to death using univariable proportional hazard model. A statistically significant shorter survival time was associated with an abnormal TS, bulbar onset, and older age. Table 3 also shows the adjusted HR (95 % CI) for time to death of the final multivariable proportional hazard model with a forward selection procedure. Age and TS were significantly and independently associated with time to death; site of onset was not selected because of the strong correlation with TS.

**Table 2:** Mean, (SD) and cut-off value of the TS in the left direction for the healthy volunteers categorized by sex, by age

Age (years)	TS (Newton) in male				TS (Newton) in female			
	*n*	mean	(SD)	Cut-off value^a^	*n*	mean	(SD)	Cut-off value^a^
14–40	23	8.01	(2.01)	3.99	24	5.48	(1.37)	2.74
41–60	16	6.53	(1.78)	2.97	17	5.04	(1.35)	2.34
61–85	19	4.47	(1.38)	1.71	20	4.49	(1.16)	2.17

^a^The cut-off value for abnormality is the mean value −2 SDs

*SD* standard deviation, *TS* tongue strength

**Table 3:** The crude HR (95 % CI) for time to death using the univariable proportional hazard model and the adjusted HR (95 % CI) of the final multivariable proportional hazard model with forward selection procedure

	*n*	Crude HR	(95 % CI)	Adjusted HR	(95 % CI)	Step
Age (years)	54	1.06	(1.02–1.10)	1.06	(1.02–1.10)	1
Time from symptoms to diagnosis (months)	54	0.97	(0.94–1.00)	–	–	
Sex						
Male	25	1.00	(reference)	–	–	
Female	29	1.25	(0.69–2.26)	–	–	
Onset site						
Spinal	35	1.00	(reference)	–	–	
Bulbar	19	2.03	(1.09–3.77)	–	–	
Bulbar symptoms						
No	28	1.00	(reference)	–	–	
Yes	26	1.41	(0.78–2.53)	–	–	
Bulbar signs						
No	34	1.00	(reference)	–	–	
Yes	20	1.41	(0.81–2.65)	–	–	
Gastrostomy						
No	28	1.00	(reference)	–	–	
Yes	26	1.60	(0.87–2.94)	–	–	
MPT						
>10 s	29	1.00	(reference)	–	–	
≤10 s	17	1.40	(0.69–2.83)	–	–	
TS						2
Normal	34	1.00	(reference)	1.00	(reference)	
Abnormal	20	2.21	(1.20–4.08)	2.60	(1.39–4.87)	

Age, onset site and TS met the criteria of *p* < 0.10 in the univariable proportional hazard model and were consequently valid for selection

*HR* hazard ratios, *CI* confidence interval, *MPT* maximum phonation time, *TS* tongue strength, – not selected

## Discussion

In this prospective study of newly diagnosed patients with ALS, an abnormal TS and age were prognostic factors for survival time independent of sex, presence of bulbar symptoms or signs, baseline MPT (regarded as an estimation of vital capacity [11, 16]), time from symptom onset to diagnosis, and time to gastrostomy placement. Bulbar onset was also associated with a poor survival, as reported previously [6]. However, a weak TS was associated with a poor survival regardless of the site of ALS symptom onset. Furthermore, a reduced TS at presentation is of more prognostic value than the presence of bulbar symptoms or signs*.* Thus, this independent non-invasive prognostic variable might be useful to stratify patients in studies with survival as primary outcome [10]. Information concerning survival is essential to these patients. In addition, knowledge that the bulbar muscles are weak might prompt a multidisciplinary ALS team to consult a speech and language therapist and a dietician, in order to prevent problems due to dysphagia. In contrast, disease progression will probably be slower in patients with dysarthria but a relatively normal TS, which would give the patient and the team time to make plans.

The relatively poor survival of patients with an abnormal TS might be because these patients are more likely to develop dysphagia, which may give rise to malnutrition, aspiration, and pulmonary infections, or because an abnormal TS in patients with spinal onset might reflect a spreading pattern of LMN involvement, which is associated with a faster progression [14].

The median age (64 years), age range (20–80 years), and onset distribution in our study population were similar to those reported in large demographic studies of ALS. This may support the external validity of our findings [7, 11, 27]. However, the survival time was longer in our study than in population-based studies, which may be because our study was conducted in a tertiary care center [5]. In this study the percentage of women is higher than in most other studies. However, multivariable analyses with selection procedure for men and for women, separately, showed that, as expected, TS is of similar importance for the prediction of survival in men and in women. Note that TS is already corrected for sex and age.

The TS measurement device was made in our laboratory, and as it was carefully calibrated, we see no reason why results would be different with another calibrated device.

In conclusion, a decreased TS at the time of diagnosis is an important independent prognostic factor for survival in patients with ALS. Objective measurement of TS can be used to assess early bulbar involvement in clinical care.
